# Elicitors directed in vitro growth and production of stevioside and other secondary metabolites in *Stevia rebaudiana* (Bertoni) Bertoni

**DOI:** 10.1038/s41598-024-65483-6

**Published:** 2024-06-26

**Authors:** Bushra Ghazal, Amna Fareed, Nisar Ahmad, Azra, Saleh H. Salmen, Mohammad Javed Ansari, Yawen Zeng, Abid Farid, Matthew A. Jenks, Abdul Qayyum

**Affiliations:** 1 Institute of Biotechnology and Genetic Engineering, The University of Agriculture Peshawar, Peshawar, 25120 Pakistan; 2 Centre for Biotechnology and Microbiology, University of Swat-19200, Swat, Pakistan; 3 Department of Plant Pathology, Amir Muhammad Khan Campus Mardan, The University of Agriculture, Peshawar, Pakistan; 4 Department of Botany and Microbiology, College of Science, King Saud University, PO Box -2455, Riyadh -11451, Saudi Arabia; 5 Department of Botany, Hindu College Moradabad (Mahatma Jyotiba Phule Rohilkhand University, Bareilly, 244001 India; 6 Biotechnology and Germplasm Resources Institute, Agricultural Biotechnology Key Laboratory of Yunnan Province/Key Laboratory of the Southwestern Crop Gene Resources and Germplasm Innovation, Ministry of Agriculture, Yunnan Academy of Agricultural Sciences, Kunming, 650205 Yunnan China; 7 Department of Entomology, The University of Haripur, Haripur, 22620 Pakistan; 8 School of Plant Sciences, College of Agriculture and Life Sciences, The University of Arizona, Tucson, AZ 85721 USA; 9 Department of Agronomy, The University of Haripur, Haripur, 22620 Pakistan

**Keywords:** Abiotic stress, Antioxidant, Growth regulators, Proline, Tissue culture, Plant reproduction, Plant stress responses

## Abstract

*Stevia rebaudiana* (stevia) is a plant in the *Asteraceae* that contains several biologically active compounds including the antidiabetic diterpene glycosides (e.g. stevioside, rebaudioside and dulcoside) that can serve as zero-calorie sugar alternatives. In this study, an elicitation strategy was applied using 5% polyethylene glycol (PEG), sodium chloride (NaCl; 50 and 100 mM) and gibberellic acid (2.0 and 4.0 mg/L GA_3_) to investigate their effect on shoot morphogenesis, and the production of phenolics, flavonoids, total soluble sugars, proline and stevioside, as well as antioxidant activity, in shoot cultures of *S. rebaudiana*. Herewith, the media supplemented with 2 mg/L and 4 mg/L GA_3_ exhibited the highest shooting response (87% and 80%). The augmentation of lower concentrations of GA_3_ (2 mg/L) in combination with 6-benzylaminopurine (BAP) resulted in the maximum mean shoot length (11.1 cm). The addition of 100 mM NaCl salts to the media led to the highest observed total phenolics content (TPC; 4.11 mg/g-DW compared to the control 0.52 mg/g-DW), total flavonoids content (TFC; 1.26 mg/g-DW) and polyphenolics concentration (5.39 mg/g-DW) in shoots cultured. However, the maximum antioxidant activity (81.8%) was observed in shoots raised in media treated with 50 mM NaCl. The application of 2 mg/L of GA_3_ resulted in the highest accumulation of proline (0.99 μg/mL) as compared to controls (0.37 μg/mL). Maximum stevioside content (71 µL/mL) was observed in cultures supplemented with 100 mM NaCl and 5% PEG, followed by the 4 mg/L GA_3_ treatment (70 µL/mL) as compared to control (60 µL/mL). Positive correlation was observed between GA_3_ and stevioside content. Notably, these two compounds are derived from a shared biochemical pathway. These results suggest that elicitation is an effective option to enhance the accumulation of steviosides and other metabolites and provides the groundwork for future industrial scale production using bioreactors.

## Introduction

*Stevia rebaudiana* (Bertoni) Bertoni (a member of the Asteraceae) is a valuable medicinal herb that produces a commercially valuable zero-caloric sugar substitute stevioside^1^. Stevioside is a natural leaf-extracted sweetener that is 300 times sweeter than standard sugar sold commercially^2^. The *Stevia* genus contains approximately 230 species, however only *S. rebaudiana* contains these zero-calorie sweetener compounds^3^.

Stevia is an indigenous species of Central and South America^4^. It was first domesticated in Japan in 1968, and the stevioside sweetener gained commercial importance as a food supplement in the 1970s^5^. Researchers in Japan successfully developed the basic process used today to extract steviosides from stevia^6,7^. Stevia is mainly cultivated in Japan, Paraguay, Taiwan, Thailand, Brazil, Malaysia, Korea, and China and consumed today worldwide. To date, no adverse effects have been reported from its use and stevia has been approved by the US Food and Drug Administration as safe dietary supplement^8^.

Stevia produces eight leaf-derived diterpene glycoside compounds with sweetening activity^7^. Stevioside and rebaudioside-A are the major steviol glycosides whereas the minor sweeteners are rebaudioside-B, C, D, E, F and dulcoside^9^. Besides these sweetening compounds, other potentially useful secondary metabolites present in stevia include various flavonoids, coumarins, cinnamic acids, phenylpropanoids and essential oils. The diterpenoid glucosides generally comprise 60–70% of all steviosides in stevia, and when compared to sugar, are 110–270 times sweeter^10^.

Several health problems in humans can be treated employing the use of stevia, such as diabetes, dental caries, obesity, and stomach infections^11^. Stevioside, a zero caloric compound synthesized in stevia leaves is considered a healthy substitute for sugar because it does not affect blood glucose^12^. The human body does not metabolize stevioside compounds, and no receptors are present for it^13^, and stevioside is antidiabetic counterbalancing *α*-cell hypersecretion^14^. Stevioside is considering a natural and safe substitute for sugar, being nontoxic, antimicrobial, and non-carcinogenic^15^.

The stevioside biosynthesis pathway is closely related to the pathway utilized in synthesis of gibberellic acid (GA_3_), an important plant growth regulator. By the action of four enzymes copalyl diphosphate synthase (CPS), kaurene synthase (KS), kaurene oxidase (KO) and kaurenoic acid hydroxylase (KAH), the precursor geranyl geranyl-diphosphate (GGDP) is converted into steviol. As an alternative route, kaurenoic acid can be converted to gibberellin by kaurenoic acid 7-oxidase rather than to steviol by KAH. Steviol is converted to stevioside (steviol glycoside) by the action of several glycosyl transferases (UGT)^16,17^.

The concentration of secondary metabolites in the leaves of stevia varies significantly in heterogenous populations. Infertility and low viability of seeds, as well as poor performance of stem cuttings used in clonal propagation, are major issues limiting the production of homogenous population of *Stevia*^18,19^. Exposure of plants grown in vitro to biotic or abiotic elicitors has been shown to modulate the biosynthetic pathways for certain plant metabolites, often releasing higher quantities of valuable metabolites in less time^20^. As stevia is a typical glycophyte, osmotic stress adversely affects its growth and the accumulation of certain of its secondary metabolites^21^.

Elicitation is an effective tool to not only activate plant defence mechanisms, but to release highly valuable secondary metabolites such as those used in the pharmaceutical, agrochemical, bio-pesticide, cosmetic, and food additive industries^22^. The study of elicitor activity plays an important role in elucidating plant defence mechanisms, as well as the regulation and prospects for enhancing the production of valuable secondary metabolites^18^. The main aim of the study reported here is to report the effect of the potential elicitors gibberellic acid GA_3_ (growth regulator), NaCl (ionic and osmotic stress), and polyethylene glycol (osmotic stress) on shoot morphogenesis, polyphenolics content, antioxidant activity, and proline and stevioside content in shoots of *Stevia rebaudiana* cultured in vitro.

## Materials and methods

### Plant collection, growth media and micropropagation of *Stevia* rebaudiana

Plant materials were provided by the Pakistan Council of Scientific and Industrial Research (PCSIR). Plants were grown in a screen house of the Institute of Biotechnology and Genetic Engineering (IBGE), The University of Agriculture, Peshawar, Pakistan. Shoot cultures were developed using basic MS media^23^ from plantlets at Plant Tissue Culture Lab, IBGE and the seeds were stored at 4°C before inoculation. After successful growth of plants in the screen house, shoot tips were selected as explants for micropropagation and exposed to multiple chemicals 2% and 5% sodium hypochlorite (bleach), 0.1% HgCl_2_, and 70% ethanol for surface sterilization. Dr. Amna Fareed identified the plant material used in this study. The study was approved by our institution, and we hereby confirm that all methods were performed in accordance with the relevant guidelines and regulations.

### Plant growth regulators

Plant growth regulators (PGRs) were selected for proliferation of shoots 6-benzylaminopurine (BAP) at 1.0 and 2.0 mg/L. Concentrations of indole 3-acetic acid alone (IAA) at 1.0 and 2.0 mg/L were also tested. Also 1-naphthaleneacetic acid (NAA) and indole-3 butyric acid (IBA) each at 0.5 mg/L was used for shoot regeneration from leaf blade explants (Table 1). Moreover, a combination of BAP and IAA (2.0 mg/L) was used to regenerate shoots organogenetically from leaf blade explants. The combination of IAA (1.0 mg/L) and BAP (2.0 mg/L) was also used. To enhance the multiplication rate, the combination of gibberellic acid (GA_3_) 0.25 mg/L and BAP 2.0 mg/L was also tested. GA_3_ in two different concentrations (2.0 and 4.0 mg/L) in combination with BAP (2.0 mg/L) was used for shoot multiplication to test their effects on steviol glycoside accumulation.

**Table 1:** Effect of various elicitors (GA, NaCl and PEG) on steviosides content in shoot cultures of *Stevia rebaudiana*.

Treatments	Steviosides content (μL/mL)
GA_3_ 2.0 mg/L	67.32 ± 0.18 b
GA_3_ 4.0 mg/L	70.38 ± 0.22 a
NaCl 50 mM	55.33 ± 0.44 d
NaCl 100 mM	71.94 ± 0.47 a
PEG 5%	71.89 ± 0.41 a
Standard meth	80.96 ± 0.22 a
Control	60.00 ± 0.34 c

Mean data was collected from triplicate experiments after 30 days of explant incubation on Murashige and Skoog media. Mean values along with common alphabets with standard deviation (± SD) are significantly different at P > 0.05.

### Shoot culture from seed-derived *stevia* plants

Explants were cultured on MS media supplemented with various plant growth regulators. The seeds were surface sterilized with 0.2% mercuric chloride for 1 min to remove surface contamination. Seeds were then washed in sterile distilled water several times. To remove excess water, seeds were placed on autoclaved filter paper. Seeds were inoculated onto MS media without plant growth regulators. After 30 days of inoculation, the leaf explant from germinated seedling was collected and transferred to medium (MS) containing PGRs in various concentrations (Table 2). The lowest shooting response was observed from leaf explants. However, as compared to lower concentrations, 2.0 mg/L BAP was found to be the most effective concentration for maximum % shooting, mean shoot length and number of shoots per explant in stevia. Therefore, in the subsequent experiments, 2.0 mg/L was supplemented in the culture media to produce the highest shoot production (along with varied concentrations of NaCl, PEG and GA_3_).

**Table 2:** Effect of various elicitors on shooting response, mean shoot length, number of shoots per explant, and on morphological features during shoot organogenesis of *Stevia rebaudiana*. Mean values with standard deviation (± SD) are significantly different at P > 0.05.

Treatments	% Shooting	Mean shoot length (cm)	No. of shoots per explant	Morphological features
MS + 2.0 mg/L BAP + PEG 5.0%	61.00 ± 2.44	4.84 ± 2.09	5.48 ± 0.42	RG/SL
MS + 2.0 mg/L BAP + NaCl 50 mM	70.03 ± 3.14	6.80 ± 1.37	9.11 ± 1.29	SG
MS + 2.0 mg/L BAP + NaCl 100 mM	57.04 ± 4.98	5.11 ± 0.72	7.68 ± 1.51	SG
MS + 2.0 mg/L BAP + 2.0 mg/L GA_3_	87.92 ± 2.76	11.08 ± 1.35	13.83 ± 0.74	HVG
MS + 2.0 mg/L BAP + 4.0 mg/L GA_3_	80.26 ± 3.48	9.24 ± 1.35	10.36 ± 0.63	VG
Control (2.0 mg/L BAP)	91.18 ± 2.28	7.20 ± 1.39	10.87 ± 1.72	NG

*RG/SL* reduced growth/stunted leaves, *SG* stunted growth, *HVG* highly vigorous growth, *VG* vigorous growth and *NG* normal growth.

### Elicitor treatments

*Stevia rebaudiana* plants grown on the MS media were subjected to 2.0 mg/L and 4.0 mg/L concentrations of GA_3,_ 5% of polyethylene glycol (PEG), and 50 mM and 100 mM of NaCl along with control containing no PGR supplements for 28 days in combination with 2.0 mg/L BAP. After 28 days plants were examined biochemically to quantify treatment effects on secondary metabolites and stevioside content.

### Sample extraction

Samples were extracted according to Storey and Jones^24^ for biochemical analysis. Methanol: chloroform: water (MCW) buffer was used for extraction in ratio of (12:5:1). For extraction, 0.2 g of plant material was frozen in liquid nitrogen, and then 2.0 mL from MCW extraction buffer was used to homogenise the stevia tissues, followed by centrifugation at 5000 rpm for 5 min. The supernatant was collected in sterile test tubes. 2.0 mL buffer was added to re-extract pellet, and the supernatant was combined with previous supernatant extract. Next, 1.5 mL chloroform and 2.0 mL water were added to the supernatant and thoroughly vortexed to obtain a uniform extract. The extract separated with methanol layer (upper) and chloroform layer (lower), and the layers separated in sterile test tubes. The upper methanol layer was used for multiple biochemical analysis.

### Estimation of total phenolics content

Total phenolic contents (TPC) were quantified according to the method of Singleton and Rossi^25^. The extraction for TPC was prepared using the method of Storey and Jones^24^. The sample was prepared by diluting in a 1:20 ratio (0.1 mL sample, 1.9 mL distilled water). Distilled water (7 mL) was added to the sample for further dilution. Here, 500 µL Foline Ciocalteu reagent was added to extract containing test tube and mixed well. After 3 min incubation, 1.5 mL of sodium carbonates solution was added and mixed using vortexing. The absorbance of the solution was measured at 760 nm after 60 min incubation. The distilled water was used as blank. The different concentrations of Gallic acid (10 µg/mL, 20 µg/mL, 30 µg/mL, 40 µg/mL, and 50 µg/mL) were used to develop a standard calibration curve.

### Estimation of total flavonoids content

The method of Balestrasse et al.^26^ was used to measure total flavonoids content. In this procedure, 2.0 mL of the sample was placed in a test tube, and 0.6 mL of NaNO_2_ was added and vortexed. Next, Al (NO_3_)_3_ (10%) of 0.5 mL, NaOH (4.3%) of 3 mL was added, and the solution vortexed again and left for 15 min. For rutin analysis 2 mg/mL (70:30) v/v ethanol–water was used.

### Determination of Antioxidant activity

The antioxidant assay was performed using methods of Miller et al.^27^ and Re et al.^28^. The plant-extracted sample of 200 mL was added to the test tube. The test tube was shaken using vortex. The absorbance of the mixture was measured at 734 nm 6 min after sample was added. Phosphate buffer saline was added 100 mL to blank 1900 µL of DPPH solution. Ascorbic acid was used as a standard for calibration. To calculate total antioxidant activity, the following formula was used:

$$TEAC = (\Delta A - B) \times F/a \times E$$

### Sugar content test

The method of Dubois et al.^29^ was used for determination of sugar content. In a sterile test tube plant sample of 1 mL was taken and 1 mL distilled water and 5 mL concentrated sulfuric acid along 1 mL (15%) phenol was added to the test tube containing plant sample. After vortexing, test tubes were placed in water bath at 25 °C for 10 min. The absorbance was measured at 490 nm. For standard different concentrations of D-glucose was used.

### Proline analysis in *stevia* tissues

Proline quantification was accomplished using methods according to Bates et al.^30^. To sample test tubes containing 1 mL of sample, 1.5 mL of glacial acetic acid and 1.5 mL of ninhydrin was added. For 45 min, sample test tubes were heated at boiling temperature. The tubes were then cooled, and 5 mL toluene was added and vortexed and then cooled for 30 min for separations. The upper toluene layer was measured for absorbance at 515 nm to determine proline amounts. L-proline was used as the standard.

### Steviosides analysis

With slight modification, stevioside was quantified using methods of Aman et al.^1^ and Dey et al.^12^. For extract preparation, dried stevia tissues treated with different elicitors was ground using mortar and pestle. A sample of 1 g was added to 10 mL of ethanol and placed on a shaker for 7–8 days. The solution was filtered after 7–8 days and dried well. The dried extract was stored in airtight bottles after weighing. 20 mg of dried extract was added and prepared in 10 mL of HPLC grade ethanol. For quantification, the Shimadzu HPLC system (LC-8A; Japan) was used, equipped with a C-18 column (150 × 4.6 mm)$,$ binary pump, 10 µL injection loop, variable length detector and solvent vacuum degasser. For mobile phase, 1.5 mL min^-1^ flow rate, and HPLC grade methanol of (70%; A) and water (30%; B) were used. In HPLC grade water (200 µg mL^−1^), a 10 µL stevioside standard (Sigma; USA) preparation µL was used. Retention time comparison was according to standard retention time and detection wavelength was 210 nm. Chromatographic analysis was performed with Clarity software, version 2.7.3.498 (2009).

### Statistical analysis

Data obtained were analyzed using analysis of variance (ANOVA) by a two-factor factorial in completely randomized design and mean comparisons (p ≤ 0.05) were carried out by Duncan Multiple Range test using M-Stat-C Statistical software.

## RESULTS

### Abiotic stress-induced shoot organogenesis in *Stevia* rebaudiana

Abiotic elicitors are commonly used in tissue culture media for enhancement of morphological characters and mass production of major medicinal secondary metabolites of commercial interest. In this study, stress inducers such as 5.0% polyethylene glycol (PEG), sodium chloride (NaCl; 50 and 100 mM) and gibberellic acid (2.0 and 4.0 mg/L GA_3_) were applied to investigate its effect on shooting frequency (%), mean shoot length, number of shoots per explant, and on other morphological characters (Table 2). Herein, 2.0 mg/L BAP augmented media was used as control for shoot organogenesis.

### Shooting response of *Stevia* rebaudiana

Media supplemented with 2.0 mg/L and 4.0 mg/L GA_3_ showed best shooting response as a function of % explants producing shoots (87% and 80%). In comparison to treatments, control media showed maximum shooting response 91%. Comparatively the control cultures containing BAP enhanced direct shoot regeneration from leaf explants. However, the augmentation of GA_3_ in combination with BAP also produced optimal shoots regeneration from leaf explants of *Stevia rebaudiana* and also improved the initiation of callus. In contrast, the addition of salt reduced the regeneration potential of shoots from leaf explants (Table 2). The PEG application also minimizes the shooting frequency (61%) as compared to control cultures (91.18%).

### Mean shoot length of *stevia* plantlets exposed to potential elicitors

PEG 5% treatment resulted in shorter shoot length (4.84 cm) as compared to control (7.20 cm) (Table 2). However, the augmentation of lower concentrations of GA_3_ (2.0 mg/L) in combination with BAP enhanced maximum mean shoot length (11.08 cm) above controls. The higher GA_3_ concentration (4.0 mg/L) with BAP combination reduced mean shoot length (9.24 cm). The shoot length was also measured when exposed to different concentration of NaCl (50 mm, 100 mM). Both the salt concentrations reduced the growth of stevia shoots. The lower salt concentration (50 mM) induced media displayed 6.80 cm mean shoot length while the higher salt concentration (100 mM) inhibited the mean shoot length of stevia to 5.11 cm (Table 2).

## Effect of potential elicitors on shoot multiplication in *Stevia* rebaudiana

The maximum number of shoots per explant was observed when the explant was exposed 2.0 mg/L GA_3_ in combination with 2.0 mg/L BAP (Table 2). However, the higher concentration of GA_3_ (4.0 mg/L) with BAP combination reduced mean number of shoots per explant (10.36) as compared to control cultures (10.87). Similarly, the higher concentration of NaCl salt (100 mM) negatively affected multiplication rate of stevia (5.11), but the lower concentration (50 mM) positively influenced the multiplication rate (6.80) as compared to control. In contrast, when leaf explants were incubated in culture medium supplemented with 5% PEG, the number of shoots per explants was highly suppressed, as shown in (Table 2).

### Morphological characteristics

**Figure 1:**
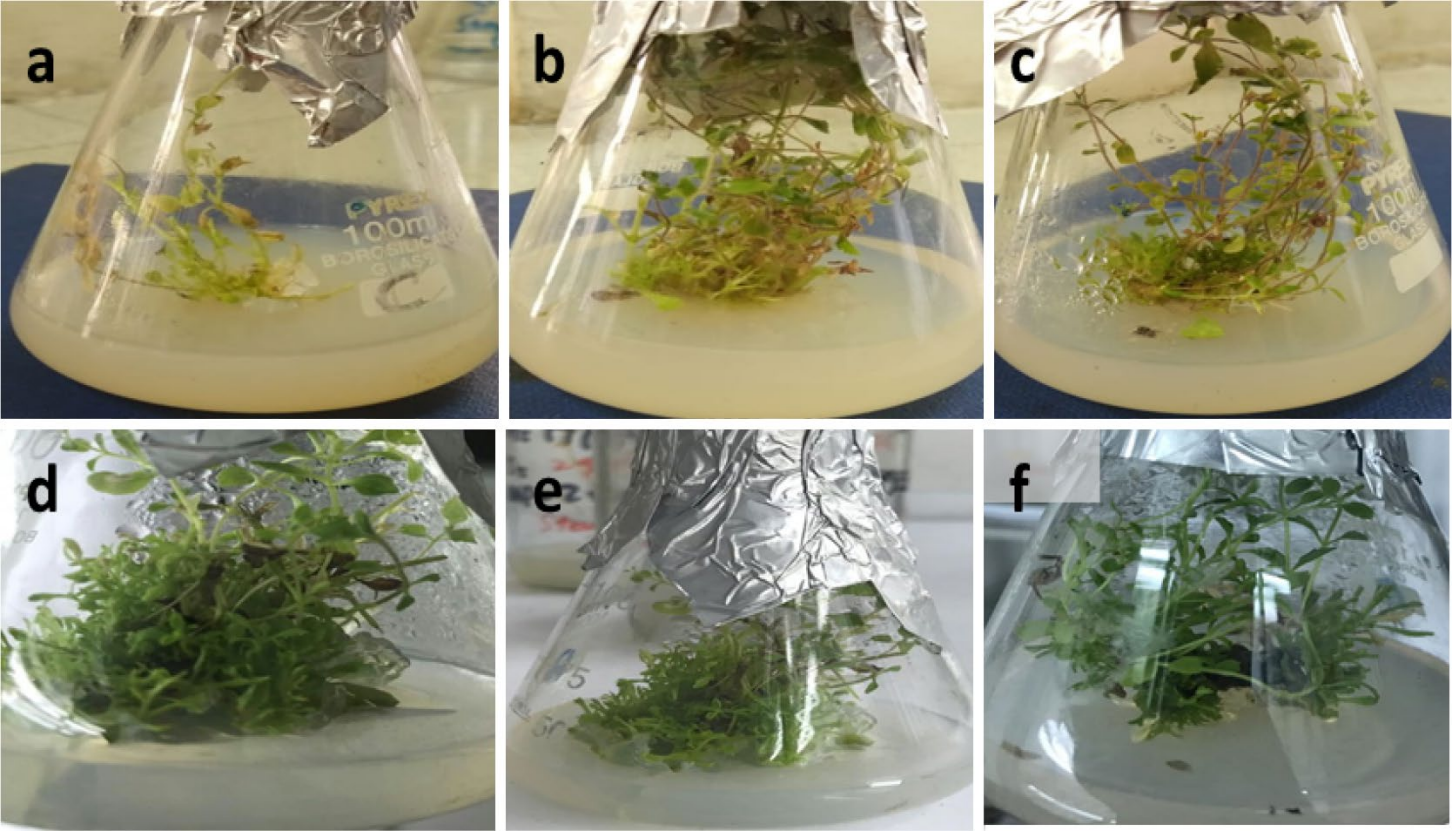
Morphological features of stevia shoots exposed to various elicitors (**a**) reduced growth and stunted leaves (**b**) stunted growth (**c**) stunted growth (**d**) highly vigorous growth (**e**) vigorous growth and (**f**) normal growth.

Morphological features displayed reduced, stunted, vigorous, and highly vigorous plant growth while using 5% PEG, NaCl (50 mM, 100 mM), GA_3_ (2.0 mg/L) and GA_3_ (4.0 mg/L) media respectively (Table 2; Fig. 1).

### Effect of potential elicitors on the production of phenolics

**Figure 2:**
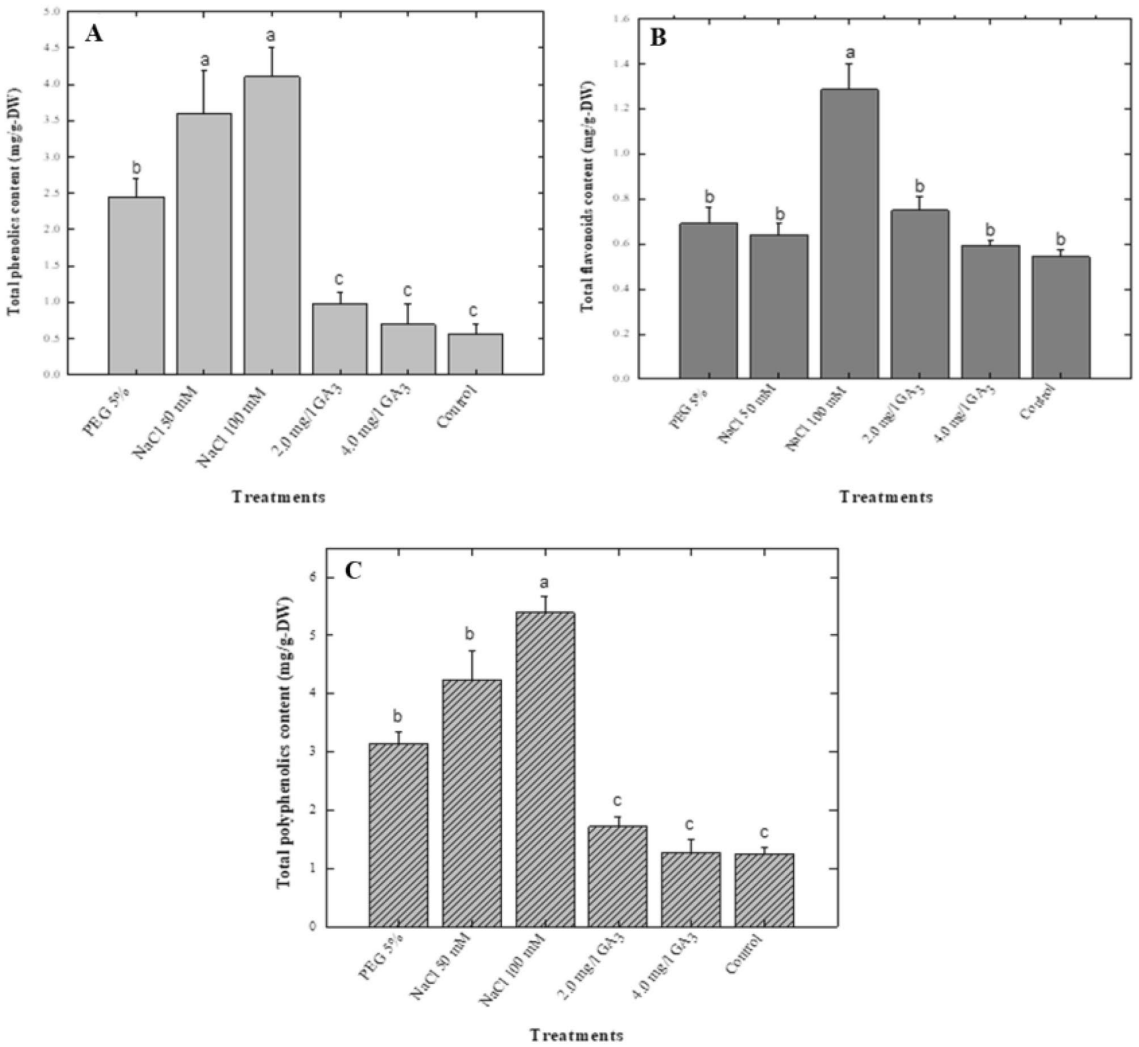
Effect of various elicitors on total phenolics (**A**), flavonoids (**B**) and polyphenolics (**C**) content in *Stevia rebaudiana.* Mean data was collected from triplicate experiments on dry weight basis. Mean values along with `same letters are not significantly different common alphabets and with standard deviation (± SD) are significantly different at *P* > *0.05.*

The total phenolic content (TPC) was enhanced with addition of NaCl to the culture medium. Highest TPC (4.11 mg/g DW) was observed when cultures were exposed to higher concentration of NaCl (100 mM), while the 50 mM NaCl-produced 3.59 mg/g-DW TPC, which was comparatively higher than control (0.52 mg/g-DW). The two different concentrations of GA_3_ produced 0.97 and 0.69 mg/g-DW phenolics content. However, the PEG treatment (5%) was also effective in increasing the biosynthesis of phenolics (Fig. 2).

### Effect of potential elicitors on production of flavonoids

Abiotic stress and GA_3_ treatments showed significant increase in flavonoid content. Minimum flavonoid content was observed in the control treatment. Similarly, like phenolics, flavonoids were also produced in the highest quantities when exposed 100 mM NaCl concentration (1.26 mg/g-DW). The addition of lower NaCl concentration produced 0.64 mg/g-DW flavonoids content (Fig. 2). Significantly, similar quantities of flavonoid amounts (0.69 mg/g-DW) were observed when cultures were exposed to PEG (5%). The culture exposures to higher GA_3_ (4.0 mg/L) enhanced TFC (0.59 mg/g DW) in comparison with control treatment (0.51 mg/g DW). The lower GA_3_ (2.0 mg/L) produced 0.75 mg/g-DW flavonoids content, which was comparatively higher to that of control cultures.

### Effect of potential elicitors on polyphenolics biosynthesis

Total phenolic content (TPC) was observed a gradual increase in in stress treatments, especially PEG (5%), NaCl (50 and 100 mM), while a reduction was observed upon exposure to GA_3_ treatments. Maximum polyphenolics (5.39 mg/g-DW) biosynthesis was observed when shoots were cultured in media containing 100 mM NaCl. The 50 mM NaCl treatment also produced the highest amount of polyphenolics (4.23 mg/g-DW) as compared to control (1.03 mg/g-DW). Comparatively, the PEG (5%) was also found to be effective in the biosynthesis of polyphenolics (3.14 mg/g-DW) as compared to control (1.03 mg/g-DW) as shown in (Fig. 2) Both GA_3_ treatments (2.0 and 4.0 mg/L) produced significantly similar quantities of polyphenolics (1.72 and 1.28 mg/g-DW).

### Correlation between phenolics and flavonoids content with total polyphenolics

**Figure 3:**
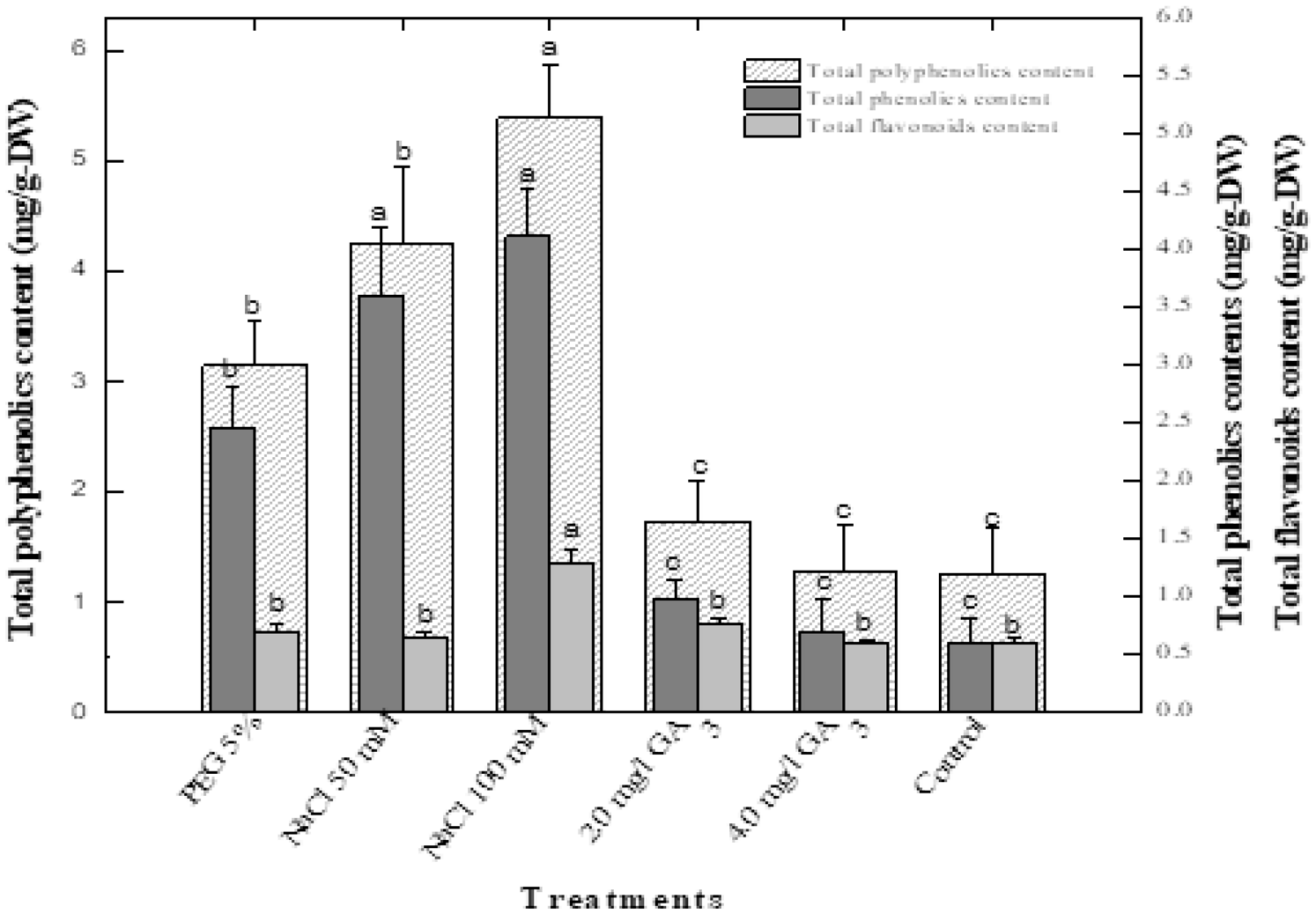
Effect of various elicitors on correlation of polyphenolics content with phenolics and flavonoids in *Stevia rebaudiana.* Mean data was collected from triplicate experiments on dry weight basis. Mean values along with common alphabets and with standard deviation (± SD) are significantly different at *P* > *0.05*.

A positive correlation was observed between total phenolic and flavonoid amounts and total polyphenolics in shoot cultures upon exposure to various stress elicitors. A significant linear correlation was observed in the biosynthesis of various metabolites when the culture media was augmented with different concentration of GA_3_ (2.0 and 4.0 mg/L). Exposure of shoots to lower doses of GA_3_ (4.0 mg/L) increased the accumulation of phenolics (0.97 mg/g DW) and flavonoids (0.75 mg/g DW) and subsequently produced higher quantities of total polyphenolics (1.73 mg/g-DW). As the concentration of the hormone was increased the production of secondary metabolites also increased. The application of 4.0 mg/L of GA_3_ reduced biosynthesis of phenolics (0.69 mg/g DW), flavonoids (0.59 mg/g-DW) and minimized the synthesis of total polyphenolics (1.28 mg/g-DW). As such, secondary metabolites production showed some dependency to NaCl concentration in Stevia in vitro shoots (Fig. 3). A linear correlation was also observed after the application of NaCl. The shoots exposed to 50 mM NaCl produced 3.59 mg/g-DW phenolics, 0.64 mg/g-DW flavonoids and produced polyphenolics in higher quantities (4.23 mg/g-DW). As the salt concentration increased the production of phenolics (4.11 mg/g-DW), flavonoids (1.28 mg/g-DW) and total polyphenolics (5.39 mg/g-DW) also increased. The 5% PEG treatment also increased the synthesis of phenolics (2.45 mg/g-DW), flavonoids (0.69 mg/g-DW) and the total polyphenolics (3.14 mg/g-DW). The combination of flavonoids and phenolics are collectively referred to as total polyphenolics in this analysis. We observed that an increase in either phenolics or flavonoids was directly proportional to the amount of polyphenolics in shoot cultures of *Stevia rebaudiana*.

### Effect of potential elicitors on antioxidant activity

**Figure 4:**
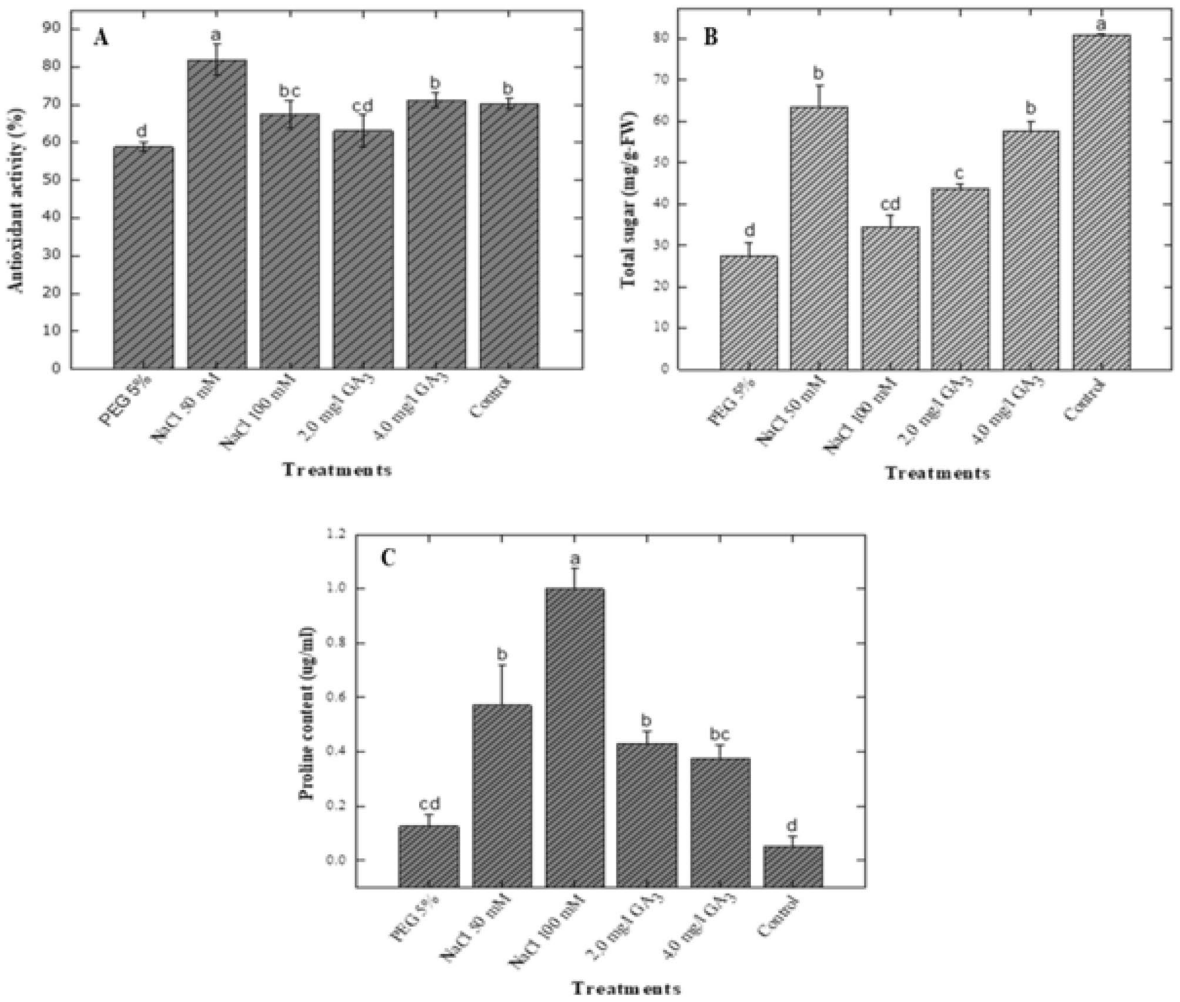
Effect of various elicitors on antioxidant activity (**A**), total soluble sugar (**B**) and proline content (**C**) in *Stevia rebaudiana.* Mean data was collected from triplicate experiments on dry weight basis. Mean values along with common alphabets and with standard deviation (± SD) are significantly different at *P* > *0.05.*

Maximum antioxidant activity (81.83%) was observed in shoots treated with NaCl (50 mM) while the higher 100 mM NaCl induced less (67.38%) antioxidant activity (Fig. 4). The control cultures and 4.0 mg/L GA_3_ augmented cultures showed the same amount of antioxidant potential (70. 28 and 71.22%). However, the lower GA_3_ (2.0 mg/L) displayed 63.05% antioxidant activity. The lowest antioxidant potential (58.79%) was observed when shoots were grown in culture media supplemented with 5% PEG inducer.

### Effect of potential elicitors on total soluble sugar

The osmotic stress inducer 5% PEG enhanced the biosynthesis of total soluble sugars (80.86 mg/g-FW) in shoot cultures of stevia as compared to control cultures (57.72 mg/g-FW) as shown in (Fig. 4). The higher NaCl concentration (100 mM) also stimulated the synthesis of soluble sugar (63.53 mg/g-FW). However, the lower NaCl concentration (50 mM) inhibited the biosynthesis of soluble sugars (27.31 mg/g-FW) in shoots in vitro. PEG caused more induction of soluble sugars in shoot cultures of stevia than NaCl treatment. GA_3_ treatments also inhibited the synthesis soluble sugars. The lower GA_3_ concentration (2.0 mg/L) displayed 34.53 mg/g-FW total soluble sugars while the higher concentration (4.0 mg/g-FW) accumulated 43.6 mg/g-FW total soluble sugars as compared to control (57.72 mg/g-FW).

### Effect of potential elicitors on proline content

The proline content in shoot cultures of stevia were investigated in response to different elicitors including PEG, NaCl and GA_3_. The lowest proline content (0.052 μg/mL) was observed when shoots were grown in culture media containing 5% PEG as compared to control (0.37 μg/mL). The application of 50 mM NaCl resulted in 0.126 μg/mL proline content. However, the addition of the higher concentration of NaCl (100 mM) to culture media enhanced proline content (0.57 μg/mL). In this study, the different concentration of GA_3_ were found to be effective for inducing higher proline content. The application of 2.0 mg/L of GA_3_ exhibited the highest accumulation of proline (0.99 μg/mL) as compared to control cultures (0.37 μg/mL). In contrast, the higher concentration of GA_3_ (4.0 mg/L) reduced the synthesis of proline content (0.43 μg/mL) as shown in Fig. 4.

### Correlation of antioxidant activity with polyphenolics in shoot cultures exposed to potential elicitors.

**Figure 5:**
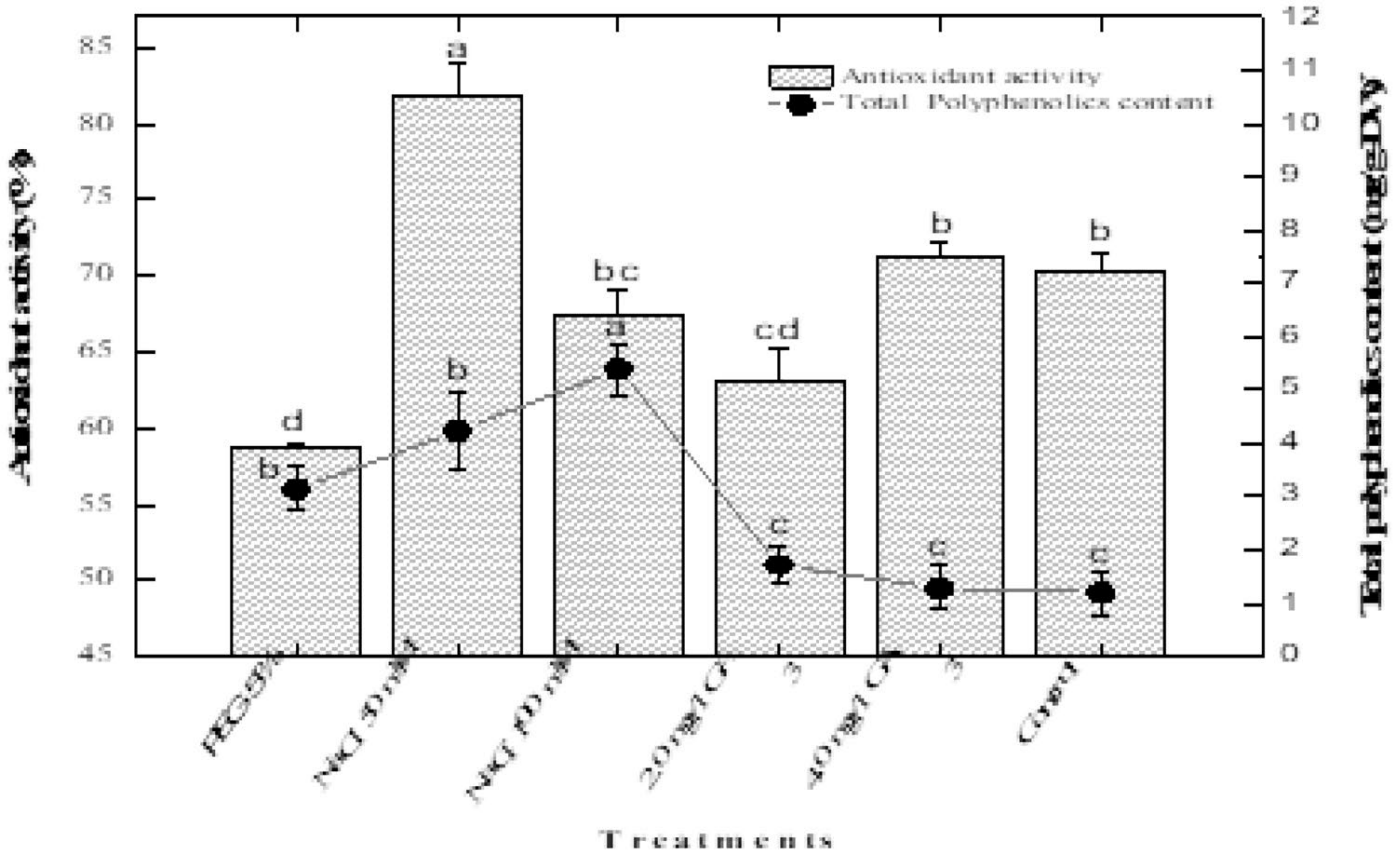
Effect of various elicitors on correlation of polyphenolics content with antioxidant activity in *Stevia rebaudiana.* Mean data was collected from triplicate experiments on dry weight basis. Mean values along with common alphabets and with standard deviation (± SD) are significantly different at *P* > *0.05.*

The effect of various stress inducers on the biosynthesis of total secondary metabolites (polyphenolics), and its correlation with antioxidant activity, was examined. Some stress inducers exhibited positive correlations between polyphenolics and antioxidant activity while other showed negative correlations. In vitro shoot cultures exposed to 2.0 m/L GA_3_ exhibited 63.05% antioxidant activity and produced 1.72 mg/g-DW total polyphenolics content (Fig. 5). In contrast, the application of the higher dose of GA_3_ (4.0 mg/L) reduced the biosynthesis of polyphenolics (1.28 mg/g-DW) but improved the antioxidant potential (71.22%). It means that the antioxidant activity and polyphenolics did not show positive and linear correlation with each other but correlated negatively. Furthermore, a similar negative correlation was observed when the shoot cultures were grown in culture media supplemented with different NaCl concentrations. The media augmented with 50 mM NaCl concentration displayed shoots having 81.83% antioxidant activity and producing 4.23 mg/g-DW of total secondary metabolites. But when the NaCl concentration increased from 50 to 100 mM, it enhanced the biosynthesis of total secondary metabolites (5.39 mg/g-DW) but reduced the antioxidant potential in shoot cultures of stevia as compared to control (1.03 mg/g-DW; 70.28%). Here, it has been observed that the NaCl and GA_3_ displayed a negative correlation between polyphenolics and antioxidant activity. Moreover, *Stevia rebaudiana* shoots exposed to 5% PEG exhibited 58.79% antioxidant activity and produced 3.14 mg/g-DW total polyphenolics in in vitro (Fig. 5).

### Quantification of diterpene glycosides (steviosides) by HPLC in *stevia* in vitro shoots exposed to potential elicitors

**Figure 6:**
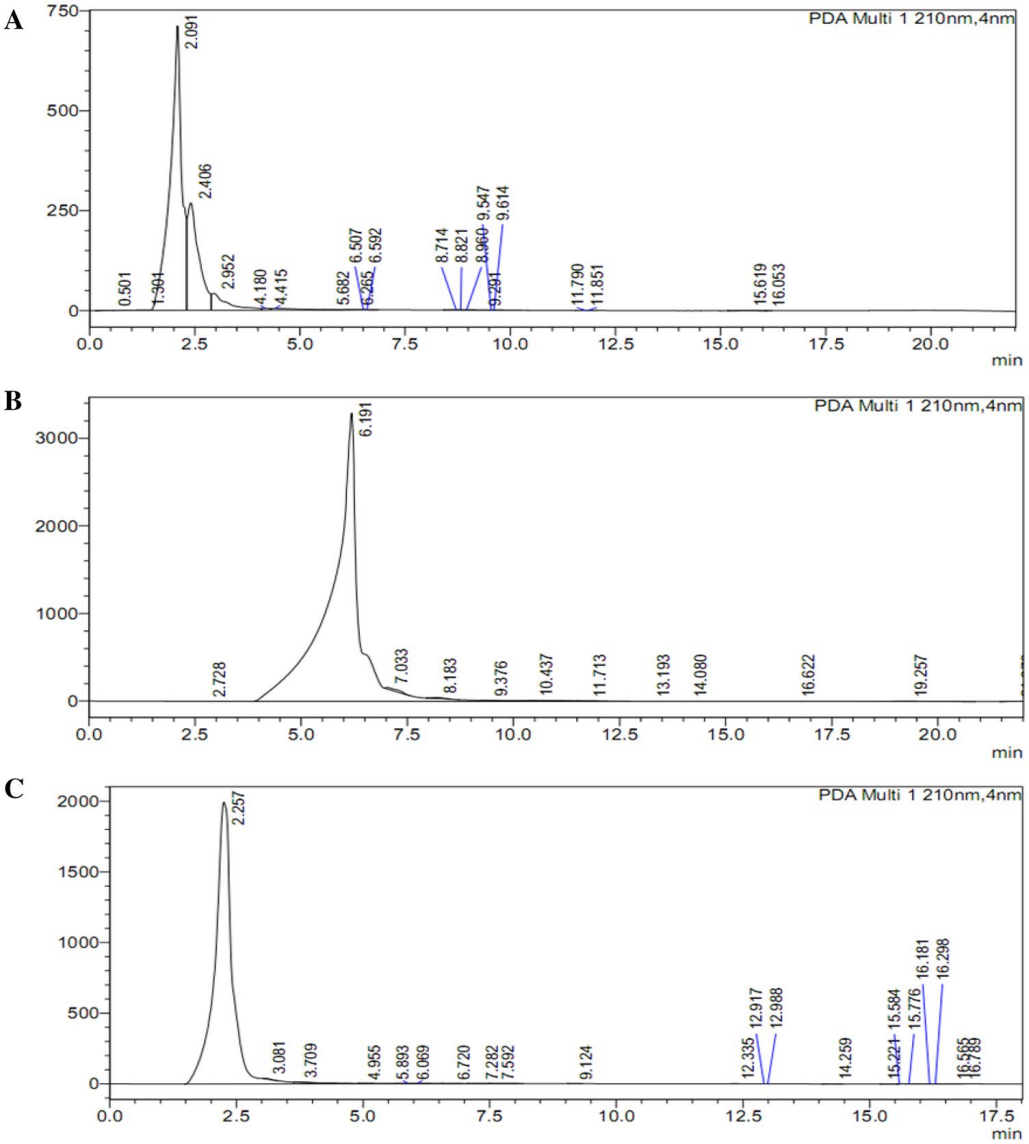
Effect of 2.0 mg/L of GA_3_, 4.0 mg/L of GA_3_ and NaCl (50 mM) on stevioside content determined using HPLC in shoot cultures of *Stevia rebaudiana.*

Exposure of plant in vitro cultures to various elicitors (GA_3_ and ionic and osmotic stressors) is one of the best strategies to enhance the biosynthesis of active secondary metabolites. The rebaudiosides and dulcosides are produced in very limited quantities in stevia leaves, whereas steviosides are typically produced in much higher amounts. Selected concentrations of GA_3_, NaCl and PEG were exploited as elicitors to enhance the synthesis of steviosides. The high-performance liquid chromatography results revealed that the addition of 2.0 mg/L GA_3_ to the in vitro shoot culture medium resulted in 63.32-μL/mL stevioside content in stevia shoots. We also observed that increased of GA_3_ concentration increased the biosynthesis of stevioside also increased from 63.32-μL/mL to 70.38-μL/mL (Table 1; Fig. 6).

**Figure 7:**
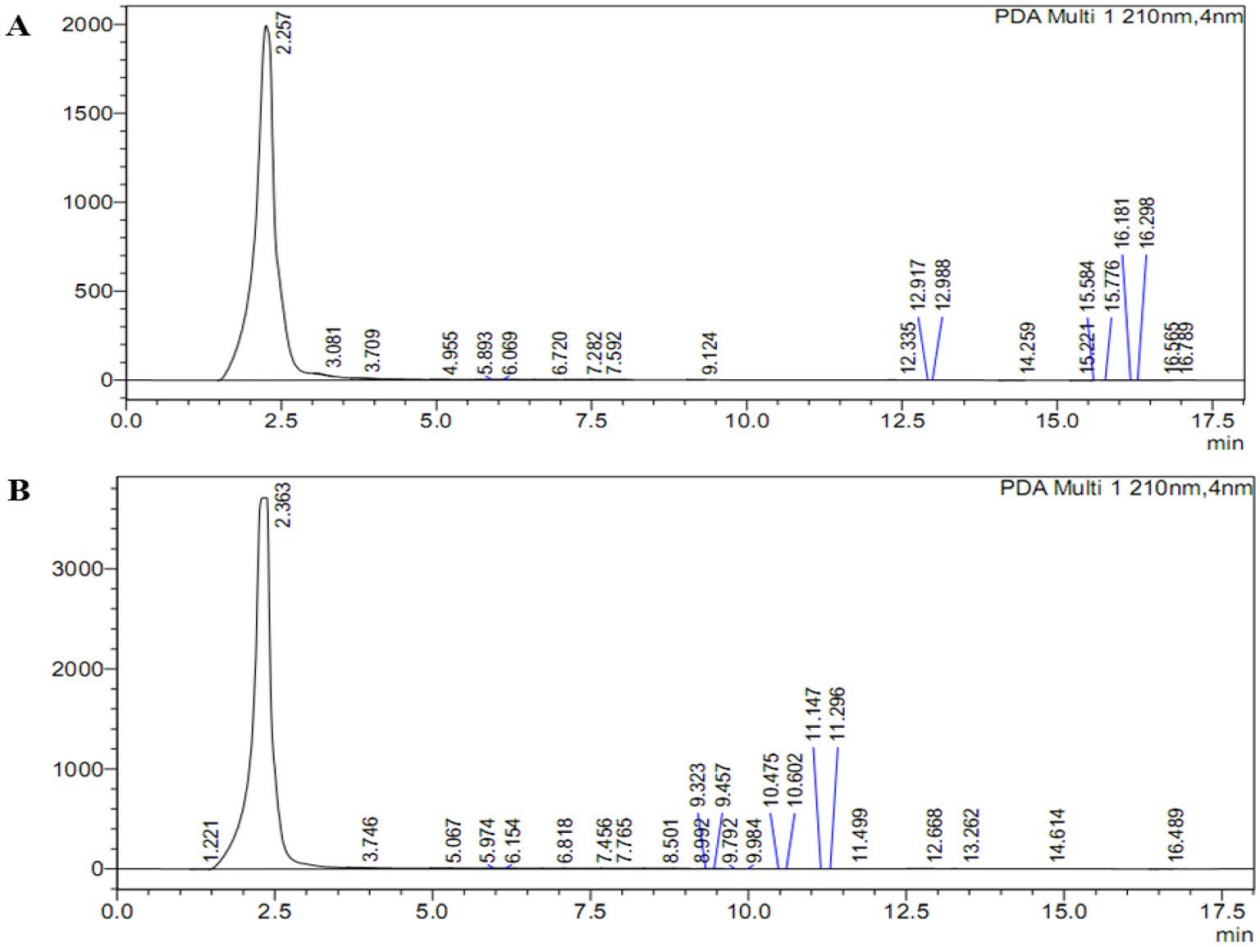
Effect of NaCl (50 mM) (**A**) and PEG (5%) (**B**) on stevioside amount determined using HPLC in shoot cultures of *Stevia rebaudiana.*

It has been reported that GA_3_ plays a central role in the biosynthesis of steviosides in various plants. The addition of higher concentrations of GA_3_ may suppress the expression of genes responsible for the synthesis of stevioside content. Furthermore, in this study, the augmentation of lower dosages (50 mM) of NaCl to shoot cultures resulted in the synthesis of 55.33-μL/mL stevioside content in vitro. The osmotic and ionic stress increases by the addition of a higher concentration of NaCl (100 mM), and it dramatically enhanced the biosynthesis of stevioside content (71.94-μL/mL) as shown in (Figs. 6 and 7). Higher concentrations of NaCl are thus very effective for increasing stevioside content in shoots cultures of stevia.

**Figure 8:**
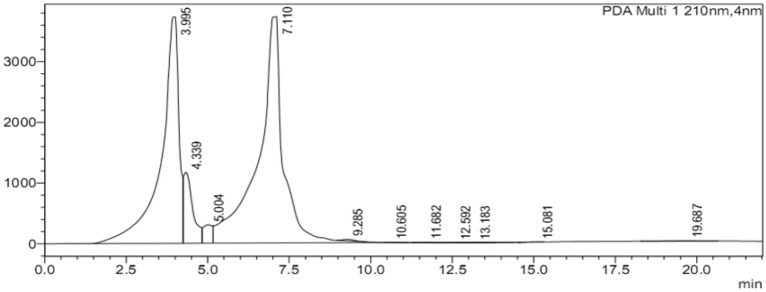
Control/standard HPLC chromatogram of stevioside.

Interestingly, a similar biosynthetic response was observed when in vitro shoot cultures of stevia were exposed to 5% polyethylene glycol elicitor. The PEG produced 71.89-μL/mL of stevioside content. In contrast, the standard solution of stevioside exhibited 80.96-μL/mL steviosides, which is comparatively greater than all treatments and control cultures (60-μL/mL) (Figs. 7 and 8). These results clearly indicated the 100 mM NaCl and PEG stressor was the best candidates for maximizing synthesis of stevioside contents in stevia in vitro shoots.

## Discussion

*Stevia rebaudiana* is one of the sweetest medicinal herbs that contains important secondary metabolites, as well as producing the sweet flavoured compound “stevioside”, a diterpene glycoside, which is 300 times sweeter than sugar. Accumulation of secondary metabolites and bioactive compounds often increase when plants are introduced to biotic and abiotic stresses^31^. To increase the production of useful metabolites in in vitro cultures, elicitation has proven to be an efficient method. Elicitors have the effect of increasing or inducing plant defence mechanisms which often involve the activation of secondary metabolism^32–34^. The goal of the current study is to determine if gibberellic acid (GA_3_), polyethylene glycol (PEG) and/or sodium chloride (NaCl) can serve as synthesis elicitors of certain antioxidant metabolites and steviosides in in vitro shoots of *Stevia rebaudiana*.

### Effect of elicitors on in vitro shoot morphogenesis

In this study the maximum shoot regeneration (87.92%) was observed in culture media containing 2 mg/L GA_3_ and 2 mg/L BAP (Table 2). The exposure of shoots to GA_3_ (2 mg/L) exhibited the highest number of shoots per explant and displayed highly vigorous growth. The least number of shoots per explant was observed in 5% PEG and the growth was stunted. Minimum shooting response was observed when shoots were exposed to 100 mM NaCl. Moreover, NaCl (50 mM) produced a 70.03% shoot induction response (Table 2). Pradhan et al. (2020) reported that among various PEG concentrations (5, 10 and 15%), the 5% PEG elicitor treatment enhanced shoot culture survival (72%) in *S. rebaudiana*. The differences in data are due to explant source and type of PEG application. Moreover, the results of Magangan et al.^35^ agree with our results where PEG and NaCl decreased growth and development of stevia seed derived plantlets. The incremental increase in PEG (2.5, 5, 7, and 10%) and NaCl (25, 50, 75, and 100 mM) concentrations reduced the growth of stevia. Similarly, Gupta et al.^36^ also reported that the addition of PEG and NaCl to the culture media displayed reduction in growth of in vitro shoots of stevia. Pazuki et al.^37^ reported that the combination of kinetin and GA_3_ promoted the growth of in vitro shoots of *S. rebaudiana*. These results are an agreement with the current study, but here we used the cytokinin BAP instead of the cytokinin kinetin. As compared to shoot cultures, Ahmad et al.^20^ exposed adventitious root cultures of stevia to various concentration of GA_3_ (0.5,1.0,1.5, and 2.0 mg/L). There results indicated a positive effect of GA_3_ on root morphogenesis and are line with the study presented here where GA_3_ improved shoot morphogenesis. It means that the application of GA_3_ not only stimulated shoot morphogenesis but also stimulated other parts of the plants as well. In this study we observed a positive response of GA_3_ on shoot organogenesis of stevia, while NaCl and PEG negatively influenced shoot morphogenesis.

### Biosynthesis of phenolics in response to elicitors in *stevia*

In the current study, PEG treatments showed a significant increase in stevia shoot phenolic content over the GA_3_ treatments and control. NaCl treatments induced the highest phenolic content of 4.11 mg/g-DW relative to all other treatments. Controls showed a minimum phenolic content of 0.52 mg/g-DW. By comparison, 2.4 mg/g-DW phenolic content was observed in PEG treatment. Ahmad et al.^38^ imposed PEG stress on nodal shoot explants of *S. rebaudiana* with concentrations of (0.5, 1, 2, and 4%). A direct correlation was observed between PEG and phenolic content. Increase in PEG concentration significantly enhanced phenolic accumulation. A 4% PEG treatment produced the highest content. Furthermore, Khan et al.^39^ induced osmotic stress with PEG concentrations of 1, 2, 3 and 4% on micro propagated *S. rebaudiana*. Treatment of 3% PEG showed maximum phenolic biosynthesis, with 4% PEG producing less phenolics. Lucho et al.^40^ exposed in vitro grown shoot tips of *S. rebaudiana* to 0.5, 1 and 1.5 g/L NaCl concentrations. Phenolic content responded similarly to NaCl concentration in our experiments, with increasing NaCl concentration and increasing phenolic accumulation. In the current study, a positive correlation of NaCl and phenolic content was observed. The highest NaCl concentration (100 mM) produced the highest phenolic content, and these results are in accordance with those of Lucho et al.^40^.

### Biosynthesis of flavonoids in response to elicitors in *stevia*

In the current study, flavonoids were produced in highest quantities (1.26 mg/g-DW) when exposed to 100 mM NaCl treatment. The lower salt concentration resulted in 0.64 mg/g-DW flavonoids in stevia shoots. Similar quantities of flavonoids were observed when cultures were exposed to PEG (5%) (Fig. 2). The lower GA_3_ (2 mg/L) produced flavonoids content slightly higher than that of the control cultures. The cultures exposed to higher GA_3_ (4 mg/L) had higher total flavonoid content as compared to control cultures (Fig. 3). Ahmad et al.^20^ reported that GA_3_ significantly increased flavonoid production (5.12 mg QE/g-DW) in adventitious root culture of *S. rebaudiana* treated with 2 mg/L of GA_3_. The combination of polyethylene glycol and paclobutrazol increased the production of flavonoids and phenolics in stevia in vitro cultures^21^. The combination of PEG along with zinc oxide enhanced flavonoid and phenolic accumulation in stevia plants. Hajihashemi and Ehsanpour^41^ also applied PEG and observed similar responses in stevia. PEG concentrations at 1, 2, 3 and 4% induced osmotic stress in micropropagated stevia which led to increased flavonoid accumulation up to 3% PEG; and then they observed declined flavonoids content at higher PEG concentrations ^39^. Another experiment conducted by Ahmad et al.^38^ where osmotic stress was imposed on shoot cultures of *S. rebaudiana* using 0.5, 1, 2 and 4% PEG concentrations wherein 4% PEG showed highest flavonoids content. Lucho et al.^41^ induced NaCl stress (0.5, 1 and 1.5 g/L) on in vitro grown shoot tips of *S. rebaudiana*. A direct relation was observed among increasing flavonoids and NaCl concentrations. Results presented here are in agreement with Lucho et al.^40^.

### Effect of elicitors on antioxidant activity

Stevia is considered a rich source of antioxidants, and leaves and callus possess strong antioxidant activity^42^. Our results revealed that NaCl (50 mM) had the highest antioxidant activity (81.83%) followed by GA_3_ 4 mg/L, control, NaCl 100 mM, GA_3_ 2 mg/L, and being lowest in 5% PEG (58.79%) treatments (Fig. 4). Most literature showed positive impact of elicitors like PEG, NaCl and GA_3_ on antioxidant activity of *Stevia rebaudiana* cultures. Adventitious root culture of *S. rebaudiana* when treated with different concentrations of GA_3_ (0.5, 1, 1.5, and 2mg/L)^20^. Highest antioxidant activity was recorded in 2 mg/L of GA_3_ treatment. Lucho et al.^40^ induced NaCl stress with various concentrations (0.0, 0.5, 1.0, 1.5g/L) on shoots tips of *S. rebaudiana*. Highest DPPH activity was observed in 1 m/L of NaCl. Results of Ahmad et al.^38^ showed that increased PEG increased DPPH activity. Various concentrations of PEG (0.5, 1.0, 2.0 and 4.0%) were imposed on in vitro grown shoots of *S. rebaudiana*. Among which 4% PEG showed maximum antioxidant activity. Khan et al.^39^ conducted same experiment with concentrations of 1, 2, 3 and 4% PEG on micropropagated *S. rebaudiana* where 3% PEG showed the highest antioxidant activity. Difference may be due to different explants of *S. rebaudiana* used in each experiment. Cantabella et al.^43^ analysed antioxidative metabolism in *ex vitro* Stevia plants by watering it with NaCl (2 and 5 g/L or 34 and 90 mM). Higher antioxidants were found in both treatments as compared to control. Here, 90 mM treatment showed high antioxidant activity than 34 mM treatment. Under certain growth conditions, the plant undergo through some stresses and produces reactive oxygen species (ROS) or free radicle which, induces deleterious effects on plants genome and deactivating the pathways needed for the growth and development of plants^44,45^. DPPH assay is most effective and reliable method of predicting antioxidant abilities of an extract or a biological source^46^.

### Effect of elicitors on total soluble sugar

In contrast to all elicitor treatments, the controls showed the highest total soluble sugar content of 80 mg/g FW (Fig. 4). In other treatments, NaCl 50 mM showed high content followed by GA_3_ 4.0, GA_3_ 2.0 mg/L, NaCl 100 mM (Fig. 4). Among all treatments the least soluble sugar content was observed in PEG treatment of 28 mg/g (Fig. 4). Results of research presented here are in agreement with Lucho et al.^40^ where shoots treated with 1 g/L NaCl expressed the highest total sugar content (108.33 eq. glucose g^-1^ DW) among other treatments (0.5 g/L, 1 g/L and 2g/L). Pot experiment on seed grown plants of *S. rebaudiana* was carried out in green house by Shahverdi et al.^47^ where plants were exposed to 30, 60, 90, 120, 150 mM NaCl concentrations in the irrigation water. Highest total soluble sugar accumulated in 30 and 60 mM NaCl (84.08 and 75.72 µg/g-FW). Higher NaCl concentrations led to decreased total soluble sugar content. Data from the current study comparable with that of Shahverdi et al.^47^.

### Effect of elicitors on proline content

Significant increase in proline content showed for NaCl and GA_3_. Highest proline content was observed in NaCl 100 mM (1.0 mg/g FW), followed by NaCl 50 mM (Fig. 4). GA_3_ concentrations also showed significant increase in proline content as compared to control and PEG (Fig. 4). GA_3_ 2.0 mg/L showed (0.42 mg/g) and GA_3_ 4.0 mg/L (0.38 mg/g) content of proline. PEG 5% showed (0.1 mg/g) proline content which is least among stress treated plants whereas least content of proline was observed in control plants less than 0.1 mg/g (Fig. 4). Azzam et al.^48^ used two varieties of *S. rebaudiana* (Sugar High A3 and Spanti). Callus of both varieties were induced by NaCl stress with different concentrations (500, 1000, 2000, 3000 mg/L). Correlation among salt and proline content was observed. Increased in NaCl concentration accumulate highest proline content. Treatment with 3000 mg/L showed highest accumulation (118.76% and 113.87 µM g^-1^). Current study is in an agreement with Azzam et al.^48^ results. When plants are under drought stress, they improve tolerance towards stress by accumulation of proline and to reduce cell injury^49^. Proline accumulation maintains cell integrity under drought stress^50^. Literature reported that drought stress increase accumulation of proline. PEG induced stress in *S. rebaudiana* micro propagation under (1, 2, 3, 4%) concentrations. Increase in stress concentration increased proline. Highest proline content (12 µM g^−1)^ was observed in 4% PEG. Least proline content was accumulated in PEG free treatment^39^. The results reported by Khan et al.^39^ and current study in case of PEG treated plants are in contrary, the variation in results may be due to explant source, plant variety and experimental procedures but NaCl treated plants are in agreement. Plant shows tolerance towards salt stress if accumulation of proline is high in leaves^51^.

### Effect of elicitors on biosynthesis of stevioside content in *stevia*

In current study, selected concentrations of GA_3_, NaCl and PEG were exploited as inducers to enhance the synthesis of steviosides. Among various elicitors, the high-performance liquid chromatography results revealed that 5% PEG and 100 mM NaCl treatments enhanced steviosides content in shoot cultures of stevia followed by GA_3_ 4.0 mg/L as compared to control. The results of Gerami et al.^52^ are in agreement with our results in case of NaCl treatment. Chitosan (0.2, 0.4, 0.6 g/L) and NaCl (50, 100, 150 mM) induced stress in green house grown stevia plants showed highest rebaudioside A content in 150 mM NaCl and 0.4 g/L chitosan. Highest stevioside content was observed in NaCl 100 mM and chitosan 4.0 g/L. The results of Lucho et al.^40^ are in contradictory where NaCl addition decreased steviosides content in comparison to control. In another study, 1.43% and 1.57% stevioside content was observed in callus when exposed to 0.10% NaCl and 0.025% Na_2_CO_3_^55^. Furthermore, Zeng et al.^53^ and Li et al.^54^ documented that the exposure of ex vitro plants to 90–120 mM NaCl promoted stevioside content (38.2%). The PEG also significantly affected the production of stevioside content in multiple cultures of Stevia. Gupta et al.^55^ and Qin et al.^56^ observed that PEG (5–10%) application reduced the biosynthesis of stevioside content in calli cultures of Stevia (1.83–1.38%). Khan et al.^39^ experiment showed maximum steviosides content (44 mg/g-DW) under 3% PEG stress. Further increase in PEG concentration by 4% showed decline in steviosides content (39 mg/g-DW). Results of Ahmad et al.^20^ are in agreement with current results where GA_3_ 2 mg/L significantly increased stevioside content 7.13 mg/g-DW in adventitious root culture of stevia. In current experiment GA_3_ 4 mg/L increased stevioside content by (70.38 mg/g-DW) in comparison to control (60 mg/g-DW). Azzam et al. ]49] irrigated seed grown *S. rebaudiana* plants by (30, 60, 90, 120, 150 mM) NaCl in green house. Maximum steviosides and rebaudioside-A was observed (8.25 and 2.82%) in 30 mM NaCl. Further increase in NaCl concentration showed a decline in stevioside and rebaudioside content. Multiple elicitors either synergistically enhanced stevioside content or antagonistically reduced the biosynthesis of Steviol glycosides. One of the objectives of the current study was to test the effect of Gibberellic acid on the biosynthesis of stevioside content. Hajihashemi and Ehsanpour^21^ studied the gene transcriptional level of six genes that are involved in the biosynthesis of stevioside and share some common pathway with Gibberellic acid. They observed that *ent-KAH*, *ent-KS1-1* and *UGT74G1* transcription was less stable upon exposure to PEG, PBZ and GA_3_. The PEG and PBZ reduced the transcriptional level, which could not be reversed by GA_3_ treatment. The GA_3_ is same precursor as *ent-kaurenoid* that is involved in the biosynthesis of diterpene glycosides along with *ent-KS* and *ent-KO*^17^. Moreover, GA_3_ treatment could not reverse the negative effect of PBZ treatment on ent-KO transcription. Ent-KO is upregulated in mature leaves where it plays a role in SVglys biosynthesis.

Stevioside and GA_3_ originate from the same biosynthetic pathway^17^, with GA_3_ arising in a one-step branch from the stevioside intermediate kaurenoic acid. In our experiment, treatment of the in vitro culture with GA_3_ (4 mg/L) increased the amounts of stevioside content. Hu et al.^57^ and Modi et al.^58^likewise showed a significant increase in leaf stevioside content by GA_3_ foliar spray. Further, Hajihashemi and Ehsanpour^21^ showed that GA_3_ supplementation to culture media resulted in an increase in both stevioside content and the transcription of six stevioside-associated genes. Whether the applied GA_3_ is directly metabolized to stevioside, or GA_3_ induces stevioside pathways indirectly, is still unknown.

## Conclusions

Our results demonstrate that BAP at 2 mg/L alone and in combination with GA_3_ at 2 mg/L induced shoot initiation, mean shoot length, number of shoots per explant and highly vigorous growth in in vitro shoot cultures of *Stevia rebaudiana*. For best shooting percentage, the GA_3_ at 2 mg/L was the most effective producing x shooting percentage. The higher concentration of NaCl (100 mM) significantly increased total phenolics, flavonoids, polyphenols and proline content comparatively to other elicitors. Lower concentration 50 mM of NaCl enhanced antioxidant activity. HPLC data revealed significant biosynthesis of important steviosides by induction of 100 mM NaCl. As GA_3_ and steviosides share common pathway a positive corelation was observed among both. This study should be expanded toward large-scale bioreactor-based production of high-valued secondary cell products of pharmaceutical importance.

## Data Availability

The datasets used and/or analyzed during the current study are available from the corresponding author on reasonable request.
